# Ciliopathies are responsible for short stature and insulin resistance: A systematic review of this clinical association regarding SOFT syndrome

**DOI:** 10.1007/s11154-024-09894-w

**Published:** 2024-07-17

**Authors:** Kevin Perge, Emilie Capel, Valérie Senée, Cécile Julier, Corinne Vigouroux, Marc Nicolino

**Affiliations:** 1 Pediatric Endocrinology, Diabetology and Metabolism Department, Femme Mère Enfant Hospital, Hospices Civils de Lyon, Bron, France; 2 Claude Bernard University, Lyon 1, Lyon, France; 3 Paris University, Institut Cochin, INSERM U1016, CNRS UMR-8104, Paris, France; 4 Sorbonne University, Inserm U938, Saint-Antoine Research Centre, Institute of Cardiometabolism and Nutrition, Paris, France; 5 Department of Endocrinology, Diabetology and Reproductive Endocrinology, Assistance Publique-Hôpitaux de Paris, Saint-Antoine University Hospital, National Reference Center for Rare Diseases of Insulin Secretion and Insulin Sensitivity (PRISIS), Paris, France; 6 Department of Molecular Biology and Genetics, Assistance Publique-Hôpitaux de Paris, Saint-Antoine University Hospital, Paris, France

**Keywords:** Ciliopathy, Insulin resistance, IGF1 resistance, Short stature, POC1A, SOFT syndrome, Adipose tissue

## Abstract

SOFT syndrome (Short stature-Onychodysplasia-Facial dysmorphism-hypoTrichosis) is a rare primordial dwarfism syndrome caused by biallelic variants in *POC1A* encoding a centriolar protein. To refine the phenotypic spectrum of SOFT syndrome, recently shown to include metabolic features, we conducted a systematic review of all published cases (19 studies, including 42 patients). The SOFT tetrad affected only 24 patients (57%), while all cases presented with short stature from birth (median height: -5.5SDS([-8.5]-[-2.8])/adult height: 132.5 cm(103.5–148)), which was most often disproportionate (90.5%), with relative macrocephaly. Bone involvement resulted in short hands and feet (100%), brachydactyly (92.5%), metaphyseal (92%) or epiphyseal (84%) anomalies, and/or sacrum/pelvis hypoplasia (58%). Serum IGF-I was increased (median IGF-I level: + 2 SDS ([-0.5]-[+ 3])). Recombinant human growth hormone (rhGH) therapy was stopped for absence/poor growth response (7/9 patients, 78%) and/or hyperglycemia (4/9 patients, 45%). Among 11 patients evaluated, 10 (91%) presented with central distribution of fat (73%), clinical (64%) and/or biological insulin resistance (IR) (100%, median HOMA-IR: 18), dyslipidemia (80%), and hepatic steatosis (100%). Glucose tolerance abnormalities affected 58% of patients aged over 10 years*.* Patients harbored biallelic missense (52.4%) or truncating (45.2%) *POC1A* variants. Biallelic null variants, affecting 36% of patients, were less frequently associated with the SOFT tetrad (33% vs 70% respectively, p = 0.027) as compared to other variants, without difference in the prevalence of metabolic abnormalities. *POC1A* should be sequenced in children with short stature, altered glucose/insulin homeostasis and/or centripetal fat distribution. In patients with SOFT syndrome, rhGH treatment is not indicated, and IR-related complications should be regularly screened and monitored.

**PROSPERO registration:** CRD42023460876.

**Supplementary Information:**

The online version contains supplementary material available at 10.1007/s11154-024-09894-w.

## Introduction

SOFT syndrome (MIM#614,813) denoting Short stature, Onychodysplasia, Facial dysmorphism, and hypoTrichosis, is a monogenic ciliopathy caused by biallelic *POC1A* variants resulting in a primordial dwarfism syndrome [1–3]. Recently, the phenotypic spectrum has been broadened to include insulin resistance (IR) and diabetes [4–10]. To date, no systematic review of the clinical features of SOFT syndrome has been conducted, and only case reports or case series with limited data on genotype–phenotype correlations were reported. In order to shed light on this novel ciliopathy, we conducted a systematic review of the literature to describe the phenotypic spectrum of *POC1A* deficiency, focusing on the characteristics of short stature and metabolic features, and identify potential genotype–phenotype correlations.

## Methods

### Inclusion criteria

We included all published studies reporting cases of SOFT syndrome due to biallelic variants in the *POC1A* gene. Cases without established molecular diagnosis or with two or more variants in at least two different genes were excluded.

### Search strategy

The literature search was carried out according to PRISMA guidelines [11], from 01/01/1970 to 31/01/2024, through Medline via PubMed, using the following [Mesh] search term: *POC1A* OR *POC1A* gene OR SOFT OR SOFT SYNDROME OR DIABETES AND *POC1A*.Other eligible studies were also searched for using the “similar articles” function in PubMed by screening the first twenty related studies of each included study. Google Scholar was then used to search for newer studies citing included studies. A subsequent search of the references of retrieved studies was also performed. Records in English, French, and Spanish language were screened. For these studies, full text versions were retrieved and independently screened (by KP, EC and CV) to determine whether they met inclusion criteria. For each included study, the full set of available data was extracted.

### Data collection

Data regarding genotypes (type and nature of variants, protein consequences and domain affected), demographic parameters (sex, last age at investigation), clinical phenotypes (neonatal growth parameters, small for gestational age, relative macrocephaly at birth, short stature, current height, onychodysplasia, facial dysmorphism, hypotrichosis, muscle cramps, high-pitched voice), clinical and paraclinical assessment of short stature (disproportionate short stature, radiological characteristics, serum IGF-I level before and during rhGH treatment, age at growth hormone (GH) onset and withdrawal, reason for stopping recombinant human GH (rhGH), height before and after rhGH treatment) and metabolic parameters (Body Mass Index [BMI] or International Obesity Task Force [IOTF], distribution of fat [evaluated clinically and/or by Dual energy X-rays absorptiometry], acanthosis nigricans, measurements of fasting and post-120-min oral glucose tolerance test (OGTT) plasma glucose and insulin, homeostasis model assessment-estimated IR [HOMA-IR] calculated as fasting glucose (mM) x insulin (mU/L)/22.5 [12], glycated hemoglobin [HbA1C], age at diagnosis of diabetes, lipid profile, hepatic transaminases, hepatic steatosis [confirmed radiologically by ultrasound and/or MRI], leptin levels) were extracted from the selected published studies. Biological IR and dyslipidemia were defined respectively by: HOMA-IR score > 2.4 and lipid panel abnormalities (LDL-chol > 160 mg/dL and/or HDL-chol < 40 mg/dL and/or triglyceridemia > 175 mg/dL) [13].

### Data and statistical analysis

Phenotype and genotype features were analyzed with descriptive statistics. The median (range) was used to summarize continuous quantitative variables, and the frequencies were used to summarize qualitative variables. Continuous quantitative variables were compared using the Student t test. Qualitative variables were compared using Fisher's Exact test. Statistical analysis was performed using the open source software R (v3.5.1., 2018, R Core Team, Vienna, Austria). Significance was determined based on p value < 0.05.

## Results

### Main characteristics of SOFT syndrome

**Fig. 1:**
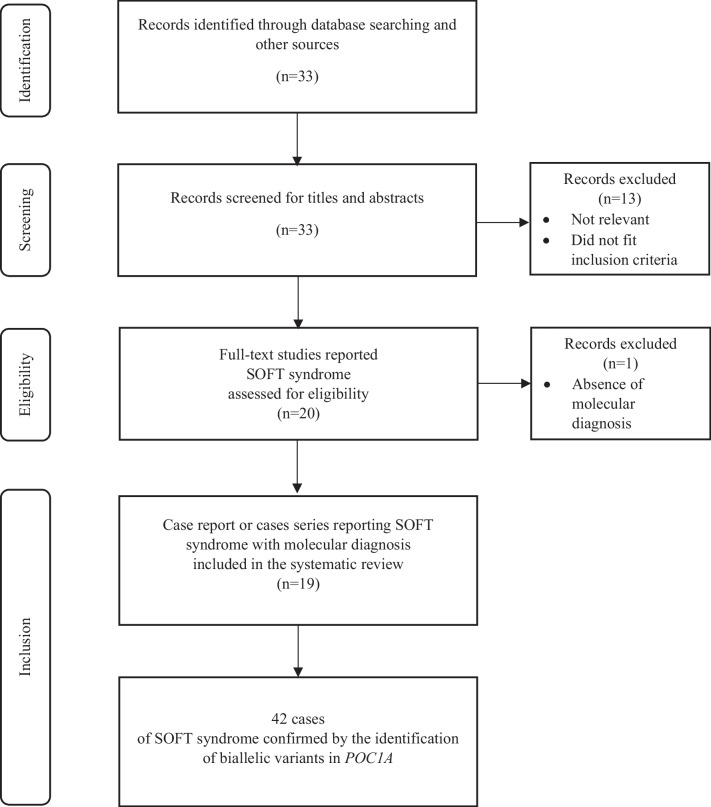
PRISMA flow diagram

A total of 33 records were identified, and a total of 19 studies were included (Fig. 1) [2–23]. These studies included a total of 42 patients issued from 27 families (including 18 consanguineous families), who presented a genetically confirmed SOFT syndrome. Tables 1 summarizes the main reported features of SOFT syndrome. Additional data for each case included are provided in Online Resource 1. Among the 42 patients with SOFT syndrome, there were 27 males and 15 females (M/F sex ratio of 1.8). The median ages at diagnosis and at last investigation were the same: 7 years (range: 0.25–42). All children were born small for gestational age (38/38) (median birth weight:-3.1 SDS (range: [-5.9]-[0]) and median birth length: -4 SDS (range: [-7.1]-[-2])). The median term at birth was 39 (range: 33–40) weeks of gestation. The majority of children were born with a relative macrocephaly (31/35, 89%) with a median birth head circumference at -1.4 SDS (range: -2.7–0.5). The tetrad “Short stature”, “Onychodysplasia”,”Facial dysmorphism” and “hypotrichosis” was present in 24 patients (57%). Regarding the short stature, present in all children with SOFT syndrome, the median height at investigation was -5.5 SDS (range: ([-8.5]-[-2.8]) and the median adult height 132.5 cm (range: 103.5–148). Onychodysplasia was the least frequent feature of the SOFT tetrad, affecting 57% of patients (24/42). All children presented with facial dysmorphism (42/42) including frontal bossing (98%), triangular and elongated face (92.5%), prominent nose (83%), dolichocephaly (46%), low-set ears (34%), hypertelorism (29%) and deep set eyes (24%). Hypotrichosis was observed in the majority of patients (31/42, i.e. 74%). All patients with onychodysplasia also presented with hypotrichosis. About half of the patients had a high-pitched voice (19/42, 45%). Muscle cramps were described in four individuals (9.5%). Approximately a quarter of patients (24%) were described with metabolic impairment: median BMI or IOTF was 23.05 (range:15–28.57), central fat distribution in eight patients, clinical and/or biological IR in 10 patients, glucose intolerance in two patients, diabetes in five patients, dyslipidemia in eight patients and fatty liver in eight patients. It should be noted that metabolic features were not systematically investigated in affected patients. Nevertheless, the prevalence of metabolic disorders reached 75% in patients aged over 10 years for those who were studied for metabolic features. Regarding neurological features, the majority of patients had normal brain development (69%). Brain MRI, performed in 12 patients, revealed anomalies of the sella turcica in three patients (n = 2: empty sella turcica /n = 1: flat sella turcica, without any reported pituitary hormone dysregulation), and vermian and/or cerebellar hypotrophy in two cases. Ophthalmological examination performed in 16 patients was abnormal in 10 of them (62.5%), with myopia (n = 2), hypermetropia (n = 3) and retinopathy (n = 5, two with reported etiology [one with diabetic retinopathy, one with bilateral macular myopic choroidosis] and three with non-reported etiology [one with mild granularity in retina and two with pigmentary retinopathy]). Regarding the hypothalamic-pituitary–gonadal axis, central precocious puberty was observed in three girls (20%—two patients were treated with Gonadotropin Releasing Hormone [GnRH] analogues), polycystic ovary syndrome in three women (20%), ovarian failure in one woman (7%) and testicular failure in one boy (4%) without further etiological data. Two patients (5%) died prematurely (unknown cause, age of death reported for one patient: 7.8 years).

**Table 1:** Main characteristics of SOFT syndrome

	**Total**	**number of missing data**
**Gender (Male, M / Female, F)**	27 M/ 15F	0
**Median (range) age at last investigation reported (years)**	7 (0.25–42)	
**Age at last investigation:**		
- < 5 years	11/33 (33.5%)	9
- 5–10 years	10/33 (30.5%)	
- 10–15 years	4/33 (12%)	
- 15–20 years	0/33 (0%)	
- > 20 years	8/33 (24%)	
**Small for gestational age**	38/38 (100%)	4
- Median (range) gestational age at birth (weeks)	39 (33–40)	27
- Median (range) birth weight (SDS)	-3.1 ([-5.9]-[0])	15
- Median (range) birth length (SDS)	-4 ([-7.1]-[-2])	18
**Relative macrocephaly at birth**	31/35 (89%)	7
- Median (range) birth head circumference (SDS)	-1.4 ([-3.3]-0)	22
**Short stature + Onychodysplasia + Facial dysmorphism + HypoTrichosis**	24/42 (57%)	0
**Short stature**	42/42 (100%)	0
- Median (range) height (SDS)	-5.5 ([-8.5]-[-2.8])	9
- Median (range) adult height (cm)	132.5 (103.5–148)	33
**Onychodysplasia**	24/42 (57%)	0
**Facial dysmorphism**	42/42 (100%)	0
- Frontal bossing	40/41 (98%)	1
- Triangular and elongated face	36/41 (92.5%)	1
- Prominent nose	34/41 (83%)	1
- Dolichocephaly	19/41 (46%)	1
- Low-set ears	14/41 (34%)	1
- Hypertelorism	12/41 (29%)	1
- Deep-set eyes	10/41 (24%)	1
**HypoTrichosis**	31/42(74%)	0
**High-pitched voice**	19/42 (45%)	0
**Muscle cramps**	4/42 (9.5%)	0
**Metabolic involvement**	10/42 (24%)	
**Metabolic involvement in patients aged over 10 years**	9/12 (75%)	
**-** Median (range) BMI (SDS)	23.05 (15–28.57)	
**-** Central distribution of fat	8/42 (19%)	32
- Clinical and/or biological insulin resistance	10/42 (24%)	
- Diabetes and/or glucose tolerance abnormalities	7/42 (17%)	
- Dyslipidemia	8/42 (19%)	
- Hepatic steatosis	8/42 (19%)	
**Neurological features:**		
- Normal psychomotor development	29/42 (69%)	0
- Empty or flat sella turcica	3/12 (25%)	30
- Vermian and cerebellar hypotrophy	2/12 (17%)	30
**Ophthalmological features**	10/16 (62.5%)	26
- Myopia	2/16 (12.5%)	26
- Hypermetropia	3/16 (19%)	26
- Retinopathy	5/16 (31%)	26
**Gonadal status**		
**-** central precocious puberty	F: 3/15 (20%) – M:0/26 (0%)	
**-** PCOS	3/15 (20%)	
- Ovarian failure	1/15 (7%)	
- Testicular failure	1/26 (4%)	
**Death**	2/42 (5%)	0

The median was used to summarize the continuous quantitative variables with extreme values (range) in parentheses. The frequencies were used to summarize the qualitative variables and were reported as "No. (%)"

*F* Female, *M* Male, *PCOS* polycystic ovary syndrome, *SDS* standard deviation score

### Genetic characteristics of SOFT syndrome (Fig. 2)

**Fig. 2:**
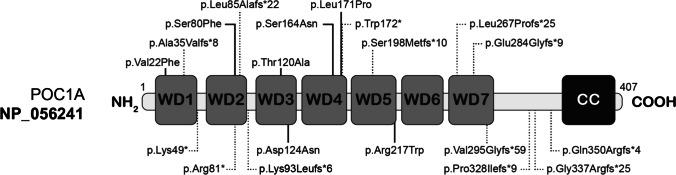
Schematic representation of POC1A protein (long isoform). The predicted localizations of *POC1A* pathogenic variants are depicted within protein sequences. N-terminal, seven β-propeller WD40 and C-terminal coiled-coil functional domains are depicted in protein sequences. The dashed lines (black) indicate truncating variants. The solid line (black) indicates non-truncating variants. The variants located below the schematic representation of POC1A protein correspond to the variants described in patients with IR and/or diabetes

SOFT syndrome is inherited in an autosomal recessive manner. The majority of the patients harbored homozygous variants (37/42, 88%). Patients harbored biallelic missense (52.4%) or truncating (45.2%) *POC1A* variants. 20 different genotypes were reported in 27 families, with 13 truncating homozygous (10/20, 50%) or compound heterozygous variants (3/20, 15%), one genotype with a truncating and a missense variant (5%), six genotypes with missense variants (five homozygous or one compound heterozygous (6/20, 30%)). A total of 21 different variants and one complete deletion were reported. The p.Arg81* variant was recurrent in six families. The p.Arg217Trp and p.Val22Phe were each present in three families. The majority of variants were exonic (17/21; seven missense and 10 nonsense), most of which affected exon 4 (4/17), although all exons could be affected. The four non-exonic variants were splicing variants. Most truncating and splicing variants are predicted to lead to the loss of one or more of the seven β-propeller WD40 repeat domains of POC1A, Three truncating variants and one splicing variant are predicted to result in the synthesis of an alternative POC1A protein deleted from its C-terminal coiled-coil domain, highly conserved upon species, which may be involved in interactions of POC1A with other proteins [24]. All the missense variants affected WD40 domains.

### Genotype/phenotype correlations

In order to study the genotype/phenotype correlations, we separated the patients into two groups according to genotype: a group of patients with ‘biallelic large truncating variants’(biallelic null variants group), i.e. biallelic splicing or truncating variants, excluding variants affecting the C-terminal end of the protein downstream of WD40 domains (n = 15, 36%), and a group of patients with ‘non-truncating or C-term truncating variants’, i.e. at least one allele with a non-truncating variant or with a truncating variant affecting the C-terminal end of the protein downstream of WD40 domains (n = 27, 64%). Indeed, it is well-known that protein-truncating variants largely result in nonsense-mediated decay preventing protein synthesis, with the exception of C-terminal truncating variants and in-frame splicing variants which can instead lead to the production of truncated protein products or proteins with in-frame deletions [25, 26]. In line with this, the expression of *POC1A* alternative transcripts and/or of truncated POC1A protein has been shown, or was likely, in patients with *POC1A* C-terminal truncating variants [4–7]. Online Resource 2 summarizes potential genotype/phenotype correlations. The SOFT complete tetrad was more frequent in the group with non-truncating or C-terminal truncating *POC1A* variants (70%) compared to the group with biallelic large truncating *POC1A* variants (33%) (p = 0.027). Regarding the facial dysmorphism, the three most frequent features (frontal bossing, triangular and elongated face and prominent nose) were equally common in both groups in contrast to low-set ears and hypertelorism which were more frequent in the group with null *POC1A* variants compared to the group with non-truncating or C-terminal truncating *POC1A* variants (p = 0.0000009 and p = 0.07, respectively). Ophthalmological features such as hypertelorism or hypermetropia, and empty or flat sella turcica were or tended to be more frequent in patients with null *POC1A* variants compared to those with missense or C-terminal truncating variants (p = 0.09, p = 0.018, p = 0.045, respectively). Conversely, high pitched voice was more frequently reported in the latter (59%) than in the former group (20%) (p = 0.023).

### Short stature: prevalence of clinical, radiological and hormonal characteristics and course of treatment with rhGH (Table 2)

**Table 2:** Short stature of SOFT syndrome: prevalence of clinical, radiological, hormonal characteristics and course of treatment with rhGH

	**Total**	**number of missing data**
**Short stature**	42/42 (100%)	0
Median (range) height at investigation (SDS)	- 5.5 (([-8.5]-[-2.8]))	9
Median (range) adult height (cm)	132.5 (103.5–148)	34
Disproportionate short stature	38/42 (90.5%)	0
Small hands and feet	40/40 (100%)	2
Brachydatyly	37/40 (92.5%)	2
Clinodactyly	11/40 (27.5%)	2
Metaphysical irregularity of long bones	24/26 (92%)	16
Hypoplasia of sacrum and pelvis	14/24 (58%)	18
Cone‑shaped epiphyses	27/32 (84%)	10
Delayed ossification of bones	14/25 (56%)	17
Stenosis of craniovertebral junction	4	?
Number of patients treated with rhGH	9/42 (21%)	0
Median (range) serum IGF-I level before rhGH treatment (SDS)	+2 ([-0.5] – [+3])	37
Median (range) age at rhGH onset (years)	6 (2.5–10.1)	33
Median (range) age at rhGH withdrawal (years)	8.7 (5.3–11.6)	33
Median (range) duration of rhGH therapy (years)	2.8 (0.66–6)	33
Reason for stopping rhGH		
- No response or weak response on growth	7/9 (78%)	33
- Metabolic cause (diabetes, excessive weight gain)	4/9 (45%)	
Median (range) maximum dose of rhGH (µg/kg/d)	67.5 (35–100)	40
Median (range) SDS of height before rhGH therapy	-6.7 ([-8.5]-[-5.5])	38
Median (range) SDS of height after rhGH treatment	-5.95 ([-8.5]-[-4.5])	38
Median (range) maximum serum level of IGF-I under rhGH treatment (SDS)	+3	41

The median was used to summarize the continuous quantitative variables with extreme values (range) in parentheses. The frequencies were used to summarize the qualitative variables and were reported as "No. (%)"

*rhGH* recombinant human Growth Hormone, *SDS* standard deviation score

Short stature was present in all reported children with SOFT syndrome. Growth retardation was very severe, with a median height of-5.5 SDS at investigation (range: ([-8.5]-[-2.8]) and a median adult height of 132.5 cm (range: 103.5–148). The majority of patients presented with bone involvement with clinically disproportionate short stature (90.5%), small hands and feet (100%), brachydactyly (92.5%) and clinodactyly (27.5%). The radiographic features included metaphysical irregularity of the long bones (92%), cone‑shaped epiphyses (84%) and hypoplasia of sacrum and pelvis (58%). A delayed bone age was reported in more than half of the patients (56%). Stenosis of the craniovertebral junction was observed in 4 patients. The median serum IGF-I level before rhGH treatment, evaluated in five patients, was + 2 SDS (range: ([-0.5]-[+ 3])). The GH provocation test revealed normal or even excessive GH response. Nine patients were treated by rhGH with a median treatment duration of 2.8 years (range: 0.66–6 years). RhGH was stopped prematurely in all of them at a median age of 8.7 years (range: 5.3–11.6 years) either for absence or poor growth response (78%) and/or for metabolic cause (diabetes, excessive weight gain) (45%). Median (range) SDS of height before and after rhGH therapy were respectively -6.7 SDS ([-5,5]-[-8,5]) and -5.95 SDS ([-4,5]-[-8,5]). These disappointing results were obtained despite high doses of rhGH, up to 100 ug/kg/d. The measurement of IGF1 circulating levels under rhGH was performed in only one patient, revealing very high IGF-1 levels (+ 3 SDS). All of these data suggest that patients with SOFT syndrome display a state of resistance to IGF-I.

Height was not significantly different according to the genotype of patients, with a median height of 125 cm (-5.8 SDS) in patients with biallelic large truncating *POC1A* variants, compared to 138 cm (-5.05 SDS) in patients with other *POC1A* mutations (Online Resource 2). Radiological abnormalities such as metaphysical irregularity of long bones were reported in all patients with null *POC1A* mutations, vs in 59% of those with other *POC1A* pathogenic variants (Online Resource 2). Conversely, clinodactyly was only reported in patients with non-truncating or C-terminal truncating *POC1A* variants, affecting 41% of them (Online Resource 2).

### Metabolic explorations reveal clinical, biological and morphological signs related to severe IR with a high risk for diabetes in patients with biallelic *POC1A* variants (Table 3)

**Table 3:** Metabolic features of SOFT syndrome

	**Total**	**number of missing data among the whole group of 42 reported patients**
**Metabolic involvement**	10/11 (91%)	31
Median (range) of BMI at investigation (kg/m^2^ or IOTF)	23.05 (15–28.57)	32
Central distribution of fat	8/11 (73%)	31
Clinical and/or biological sign of insulin resistance	10/11 (91%)	31
Acanthosis nigricans	7/11 (64%)	31
Median (range) fasting blood glucose (mmol/L)	5.1 (4–15.7)	33
Median (range) fasting insulinemia (mUI/L)	57.1 (12.96–241.2)	
Median (range) HOMA-IR	18 (2.9–54.6)	35
Median (range) OGTT-120 min blood glucose (mmol/L)/	8.75 (6.8–25.3)	34
Median (range) OGTT-120 min insulinemia (mUI/L)	449 (62–1818)	
Median (range) HbA1C	5.6 (5–9.6)	37
Glucose tolerance abnormality and/or diabetes		
- In all children	7/42 (17%)	0
- In children aged over 10 years	7/12 (58%)	0
Diabetes		
- In all children	5/42 (12%)	0
- In children aged over 10 years	5/12 (42%)	0
Dyslipidemia	8/10 (80%)	32
Hypertriglyceridemia	4/6 (67%)	36
Hepatic steatosis	8/8 (100%)	34
Hepatic transaminases (AST- ALT, times higher than N)	2 (2–3)	35

The median was used to summarize the continuous quantitative variables with extreme values (range) in parentheses. The frequencies were used to summarize the qualitative variables and were reported as "No. (%)"

Some qualitative variables were compared using Fisher's Exact test. Significance was determined based on p value < 0.05

Biological IR and dyslipidemia were defined respectively by: HOMA-IR score > 2.4 and lipid panel abnormalities (LDL-chol > 160 mg/dL and/or HDL-chol < 40 mg/dL and/or triglyceridemia > 175 mg/dL) [13]

*BMI* Body Mass Index, *HOMA-IR* homeostasis model assessment-estimated IR, *IOTF* International Obesity Task Force (IOTF), *OGTT* oral glucose tolerance test, *SDS* standard deviation score

11 patients were evaluated regarding distribution of fat, presence of acanthosis nigricans, glucose and insulin homeostasis, circulating lipids, and hepatic steatosis. Among them, 10 patients (91%) presented with metabolic abnormalities. Median BMI was 23.05 kg/m^2^ (range:15–28.57). Only three patients were overweight and none was obese. Most patients evaluated presented with central distribution of fat (8/11,73%, 3 confirmed by Dual energy X-rays absorptiometry). 10 patients (91%) presented with clinical (7/11, 64%) and/or biological IR (7/7,100%). HOMA-IR was increased in all patients evaluated with a median of 18 (range: 2.9–54.6). OGTT performed in eight patients, revealed in five of them glucose tolerance abnormalities (n = 2) or diabetes (n = 3). Median OGTT-120 min blood glucose and insulin were 8.75 mmol/L (range: 6.8–25.3) and 449 mUI/L (range: 62–1818), respectively. Median HbA1C was 5.6% (range 5–9.6). A total of five patients were diagnosed with diabetes, at a median age of 21,5 years (range: 9.75 to 32 years). The prevalence of glucose tolerance abnormalities and diabetes was respectively 17% and 12% among all patients, but rose to 58% and 42% in patients over 10 years old. Glucose tolerance abnormalities was more frequently reported in patients treated with rhGH (4/9, 44%) as compared to non-treated patients (3/33, 9%, p = 0.02), and in females (5/15, 33%) as compared to males (2/27, 7.5%) (p = 0.07). No significant difference in the prevalence of altered glucose or insulin homeostasis was observed with respect to two groups of genotypes (Online Resource 2), and IR and diabetes were present in patients carrying *POC1A* biallelic variants regardless of their position (Fig. 2). Dyslipidemia was reported in 80% of the patients investigated (8/10), with hypertriglyceridemia in 67% of them (4/6). Hepatic steatosis was diagnosed in all the patients evaluated (8/8,100%) associated with increased hepatic transaminases (median 2N [2 times the normal range] (2–3)).

## Discussion

Our study constitutes the first systematic review of the phenotype/genotype features of SOFT syndrome. In order to shed light on the recently described metabolic and endocrinological features of this novel ciliopathy, we focused in this discussion on the characteristics of short stature and the high risk of severe IR and diabetes.

### Short stature and skeletal features: typical features of SOFT syndrome resulting from multiple mechanisms

This systematic study confirmed that the severe short stature was a major feature of SOFT syndrome related to biallelic *POC1A* variants, present in all affected individuals described to date. SOFT syndrome is a rare primordial dwarfism [1–3], with short stature present from birth, most often associated with relative macrocephaly. This association may suggest other causes of short stature with relative macrocephaly such as Russell-Silver syndrome, Mulibrey nanism, Temple syndrome, Floating Harbor syndrome and 3 M syndrome [27]. There are numerous overlapping dysmorphic findings between these syndromes, such as triangular face, dolichocephaly, or frontal bossing [27]. However, limb asymmetry is a characteristic feature of Russell-Silver syndrome, yellow dots on eye fundus and pericardial constriction in Mulibrey nanism, and a fleshy tipped nose characterizes 3 M syndrome, as summarized by Koparir et al. [14]. Prominent nose and hypotrichosis appear to be the most specific dysmorphic features of SOFT syndrome. However, due to clinical overlap, it is recommended, in patients with syndromic short stature, to perform a multigene panel testing which should systematically include the analysis of *POC1A* [27].

The underlying mechanisms of short stature may be multiple. Studies in animal models with *POC1A* deficiency revealed defects in chondrocyte proliferation and survival affecting the growth plate of long bones. The defective ciliary function in chondrocytes, responsible for multipolar spindle formation, may result in their inability to proliferate and to maintain the flattened shape required to form a cellular column after cell division [14, 28]. Clinically, the bony component of SOFT syndrome results in disproportionate short stature, small hands and feet and brachydactyly, reported in the majority of patients. These features are responsible for short arm span, an essential element to look for in children with short stature. However, precise measurements of arm span, along with sitting height and extremity lengths, are lacking in the literature data. Clinodactyly, observed in more than 2/3 of cases in patients with Russell-Silver syndrome, is less frequent in SOFT syndrome, affecting less than 1/3 of cases [29]. The presence of clinical signs pointing towards bone damage should prompt clinicians to perform whole body radiographs which may reveal metaphysical irregularity of the long bones, conical epiphyses and hypoplasia of the sacrum and pelvis. As these radiological signs are not specific for SOFT syndrome, genetic investigations should include the analysis of the *POC1A* gene in patients with short stature and skeletal involvement. Furthermore, these radiological signs should lead to search for craniovertebral junction stenosis, observed in a few patients with SOFT syndrome in this systematic review. This has an important practical consequence since craniovertebral junction stenosis may be responsible for sleep apnea syndrome, as reported in other causes of dwarfism such as achondroplasia [30]. We thus recommend to perform MRI of the whole vertebral column in early childhood in patients with SOFT syndrome. A state of resistance to IGF1, as attested by high IGF1 serum levels in several patients with SOFT syndrome, may also contribute to growth retardation. Our previous study revealed that fibroblasts from patients with biallelic *POC1A* variants showed impaired IGF1 signaling, which may arise from altered subcellular localization of IGF1 receptors secondary to the alteration of centrosome/basal body organization and ciliogenesis [10]. Interestingly, as it is the case in most children born small for gestational age, the onset of puberty may occur early in children with SOFT syndrome and may progress rapidly, decreasing the expected adult height [31, 32]. The expected benefit of GnRH analogues on the final height could not be evaluated in the two patients treated in the context of central precocious puberty due to absence of data on pre-treatment and final heights. As regards the ovarian and testicular insufficiency affecting two patients, attention should be paid to gonadal follow-up in order to better understand this potential associated impairment. Although further studies in humans are needed to elucidate the function of POC1A in endochondral ossification and growth, both defects in chondrocyte proliferation and in response to IGF1 may contribute to the poor response to rhGH therapy, reported in several studies [8, 10, 15–17, 21]. Moreover, rhGH therapy was shown to worsen IR and precipitate diabetes in several patients [8, 10]. Thus, rhGH treatment is not indicated in SOFT syndrome and should even be avoided. Knowledge of this risk should encourage clinicians to be cautious about initiating rhGH treatment in syndromic intra-uterine growth retardation. In terms of genotype and phenotype correlation, patients with a more deleterious genotype (biallelic large truncating POC1A variants) tended to have a more severe short stature compared to patients with other POC1A mutations, although the difference was not statistically significant. This non-significant finding may be partly explained by low statistical power of the test due to the small number of subjects (N = 33) and missing data. Further study is needed to conclude on the potential influence of *POC1A* genotypes on the extent of growth failure and/or on specific skeletal abnormalities or dysmorphic features.

### Metabolic disorders with severe IR and high risk of diabetes: an important feature of SOFT syndrome justifying specific monitoring of patients

This study confirms that severe IR is a prominent feature of SOFT syndrome related to biallelic *POC1A* variants. IR was present in approximately a quarter of patients in this systematic review. The prevalence of IR and/or hyperglycemia was higher in patients aged over 10 years and was found almost invariably in children who were evaluated. Thus, the prevalence of IR in SOFT syndrome would likely be even higher if patients were systematically screened from childhood. We thus recommend to perform fasting insulin/glucose measurements, and OGTT in case of normal fasting measurements in all patients with SOFT syndrome as soon as they are diagnosed, even before puberty. SOFT syndrome-associated IR, frequently severe (median HOMA-IR 18) developed progressively from childhood to adulthood, with a significant risk of diabetes, especially after puberty, increased by rhGH treatment [4–10]. Contrary to a previous hypothesis stating that the phenotype of severe IR associated with SOFT syndrome could be linked to frameshift *POC1A* variants affecting exon 10 [4–6], IR was present regardless of the position of variants in this systematic review [7–10]. Several elements point to adipocyte dysfunction as the main underlying mechanism of IR and diabetes in SOFT syndrome. Using *POC1A*-deleted human adipose stem cells, we have shown that the lack of POC1A protein expression impairs adipocyte differentiation, and induces cellular senescence [10], which are well-known mechanisms leading to lipodystrophic diseases with IR, hypertriglyceridemia and hepatic steatosis [33]. In line, our current study shows that, when adipose tissue body distribution was investigated in patients with SOFT syndrome, a phenotype of partial lipodystrophy was reported in most of them, with a paucity of body fat in limbs and a central accumulation of fat, associated with IR, hypertriglyceridemia and hepatic steatosis. Interestingly, adipocyte dysfunction has also been shown in other monogenic ciliopathies, such as Alström and Bardet-Biedl syndromes [3, 34–37], and lipodystrophic features are part of the primordial dwarfism syndrome due to pathogenic variants in *PCNT*, encoding the centrosomal protein pericentrin [38, 39]. This confirms that a proper function of centrosomes and cilia is required for the physiological development of adipose tissue, as shown in several *in vitro* studies of adipogenesis [40, 41]. In addition, POC1A dysfunction may alter the cilia-mediated recruitment of proteins at the plasma membrane, leading to the mislocalization of insulin and IGF1 receptors, which may reinforce defects in adipocyte differentiation and function [10, 42, 43]. Similarly, in Bardet-Biedl syndrome, defects in the BBSome complex could lead to abnormal cellular localization of insulin receptor, as well as proteins involved in satiety regulation [44–47].

Our current study confirms that SOFT syndrome should be considered as a lipodystrophic syndrome, although most patients have been misdiagnosed with type 2 diabetes. This confusion between type 2 diabetes and specific causes of diabetes is often reported in other monogenic causes of diabetes such as MODY [48–50]. In addition, low birth weight is a risk factor for metabolic syndrome later in life, whatever the cause, and may contribute to the metabolic disorders in these patients [51, 52]. However, IR and dyslipidemia described in patients born small for gestational age are not as severe as in SOFT syndrome [52]. Surprisingly, we observed a higher prevalence of glucose tolerance abnormalities in females than in males affected with SOFT syndrome. As proposed by numerous authors, weight gain or IR could be related to the early manifestations of puberty or adrenarche, more frequent in girls [31, 53, 54]. Similarly, polycystic ovary syndrome, which is strongly associated with IR, displays an increased prevalence in females with low birth weight and premature pubarche [55].

Finally, a limitation of this review is that we had almost no information on the metabolic phenotype of family members of index cases, making it impossible to assess the potential metabolic effects of monoallelic *POC1A* pathogenic variants, and to compare the metabolic phenotypes according to family history.

## Conclusion and practical implications

This systematic review illustrates that the establishment of a precise diagnosis in children with syndromic short stature is important to establish a correct prognosis, adapt therapeutic decisions, and provide an accurate genetic counseling to affected patients and their families. The analysis of the *POC1A* gene should be carried out in any patient with short stature with relative macrocephaly or skeletal anomalies, and prior to rhGH treatment in patients with syndromic intra-uterine growth retardation. RhGH treatment is not recommended in SOFT syndrome and should even be avoided because of its poor efficacy and the risk of worsening IR and precipitating diabetes. Severe IR is an important feature in patients with SOFT syndrome. A wider awareness of this metabolic feature is necessary to set up annual clinical and biological monitoring (HbA1C associated with an OGTT) of patients with SOFT syndrome, even before puberty, in order to detect IR and/or glucose tolerance abnormalities as soon as possible. Knowledge of this metabolic features should encourage clinicians to prescribe measures promoting healthy diet and physical activity to patients and their families from an early age. *POC1A* should also be considered as a gene involved in monogenic lipodystrophy, IR and/or diabetes in children with short stature. Finally, this study identified possible genotype/phenotype correlations, requiring confirmation in larger cohorts.

## Supplementary Information

Below is the link to the electronic supplementary material.

Online Resource 1: Set of characteristics of all patients with biallelic pathogenic *POC1A* variants reported in the literature. Footnotes: The variants are described according to the same reference sequence (NM_015426). Online Resource abbreviations: *F* Female, *M* Male, *N* normal (2N: 2 times higher than normal), *rhGH* recombinant human Growth Hormone, *SDS* standard deviation score (XLSX 39 KB)

Online Resource 2: Comparison of the characteristics of SOFT syndrome according to the severity of the mutations. Footnotes: The median was used to summarize the continuous quantitative variables with extreme values (range) in parentheses. The frequencies were used to summarize the qualitative variables and were reported as "No. (%)". Online Resource abbreviations: *Continuous quantitative variables were compared using the Student t test. Qualitative variables were compared using Fisher's Exact test. Statistical analysis was performed using the open source software R (v3.5.1., 2018, R Core Team, Vienna, Austria). Significance was determined based on p value < 0.05. *F* Female, *M* Male, *N* normal (2N: 2 times higher than normal), *rhGH* recombinant human Growth Hormone, *SDS* standard deviation score (DOCX 25 KB)

## Data Availability

No datasets were generated or analysed during the current study.
