# Supplementary material for: Ciliopathies are responsible for short stature and insulin resistance: A systematic review of this clinical association regarding SOFT syndrome
Source: Rev Endocr Metab Disord. 2024 Jul 17;25(5):827–38. doi: 10.1007/s11154-024-09894-w (PMC11470920; doi:10.1007/s11154-024-09894-w)
Supplement: Supplementary file 2 — Online Resource 2: Comparison of the characteristics of SOFT syndrome according to the severity of the mutations. Footnotes: The median was used to summarize the continuous quantitative variables with extreme values (range) in parentheses. The frequencies were used to summarize the qualitative variables and were reported as "No. (%)". Online Resource abbreviations: *Continuous quantitative variables were compared using the Student t test. Qualitative variables were compared using Fisher's Exact test. Statistical analysis was performed using the open source software R (v3.5.1., 2018, R Core Team, Vienna, Austria). Significance was determined based on p value < 0.05. F Female, M Male, N normal (2N: 2 times higher than normal), rhGH recombinant human Growth Hormone, SDS standard deviation score (DOCX 25 KB) [file 11154_2024_9894_MOESM2_ESM.docx]

**Online Resource 2:** **Comparison of the characteristics of SOFT syndrome according to the type of mutations**

|  | **Biallelic large truncating variants**  **(n=15)** | **Non-truncating or C-term truncating variants**  **(n=27)** | **Statistical analysis*** |
| --- | --- | --- | --- |
| **Gender (****Male, M / Female, F)** | 10/5 | 17/10 | - |
| **Median (range) age at last investigation reported (years)** | 6 (0.25-32) | 7.4 (2-42) | 0.48 |
| **Small for gestational age**  - Median (range) gestational age at birth (weeks)  - Median (range) birth weight (SDS)  - Median (range) birth length (SDS)  **Relative macrocephaly at birth**  - Median (range) birth head circumference (SDS) | 14/14 (100%)  38 (33-40)  -2.89 ([-5.9]-0)  -4 ([-7.1]-[-3])  10/12  -1.23 ([-3.3-]-[-0.5]) | 24/24 (100%)  39.5 (35-40)  -3.1 ([-4.4]-[-2])  -4.05 ([-5.7]-[-2])  21/23  -1.6 ([-2.7]-0) | 1  0.43  0.84  0.42  0.59  0.88 |
| **Short stature + Onychodysplasia + Facial dysmorphism + HypoTrichosis** | 5/15 (33%) | 19/27 (70%) | 0.027* |
| **Short stature**  - Median (range) current height (SDS)  - Median (range) adult height (cm)  - Disproportionate short stature  - Small hands and feet  - Brachydactyly  - Clinodactyly  - Metaphysical irregularity of long bones  - Hypoplasia of sacrum and pelvis  - Cone‑shaped epiphyses  - Delayed ossification of bones | 15/15 (100%)  -5.8 ([-8.5]- [-4.34])  125 (103.5-138)  15/15 (100%)  14/14 (100%)  12/13 (92%)  0/14 (0%)  8/8 (100%)  2/7 (29%)  7/8 (88%)  3/7 (43%) | 27/27 (100%)  -5.05 ([-7.9]- [-2.8])  138 (120-148)  23/27 (85%)  26/27 (96%)  25/27 (93%)  11/27 (41%)  16/27 (59%)  12/27 (44%)  20/27 (74%)  11/27 (41%) | 1  0.14  0.35  0.27  1  0.96  0.007*  0.037*  0.67  0.64  1 |
| **Onychodysplasia** | 5/15 (33%) | 19/27 (70%) | 0.21 |
| **Facial dysmorphism**  - Frontal bossing  - Triangular and elongated face  - Prominent nose  - Dolichocephaly  - Low-set ears  - Hypertelorism  - Deep-set eyes | 15/15 (100%)  14/14 (100%)  12/14 (86%)  12/14 (86%)  8/14 (57%)  12/14 (86%)  7/14 (50%)  5/14 (36%) | 27/27 (100%)  26/27 (96%)  24/27 (89%)  22/27 (81%)  11/27 (41%)  2/27 (7%)  5/27 (19%)  5/27 (19%) | 1  1  0.76  1  0.34  0.0000009*  0.07  0.26 |
| **HypoTrichosis** | 7/15 (47%) | 24/27 (89%) | 0.117 |
| **High pitched voice** | 3/15 (20%) | 16/27 (59%) | 0.023* |
| **Muscle cramps** | 2/15 (13%) | 2/27 (7%) | 0.60 |
| **Metabolic involvement**  Median (range) of current BMI (kg/m2 or IOTF)  Central distribution of fat  Clinical and/or biological sign of insulin resistance  Median (range) HOMA-IR  Glucose tolerance abnormalities and/or diabetes  - In all children  - In children aged over 10 years  Diabetes  - In all children  - In children aged over 10 years  Age at onset of diabetes  Dyslipidemia  Hypertriglyceridemia  Hepatic steatosis | 4/15  17 (15-28.57)  4/5  4/5  18 (4.76-29.6)  4/15 (27%)  4/5 (80%)  3/15  3/5  22 (10-32)  3/4  1/2  3/3 | 6/27  24.1 (16.5-25.5)  4/6  6/6  19.84 (2.9-54.6)  3/27 (11%)  3/7 (43%)  2/27  2/7  17.75 (14-21.5)  5/6  3/4  5/5 | 1  0.51  1  0.45  0.63  0.67  0.29  0.27  0.55  0.67  0.63  0.22  1 |
| **Neurological features :**  - Normal psychomotor development  - Empty or flat sella turcica  - Vermian and cerebellar hypotrophy | 10/15  3/5  1/5 | 19/27  0/7  1/7 | 0.84  0.045*  1 |
| **Ophthalmological features**  - Myopia  - Hypermetropia  - Retinopathy | 5/5 (100%)  0/5  3/5  2/5 | 5/11 (45%)  2/11  0/11  3/11 | 0.09*  1  0.018*  1 |
| **Gonadal status**  **-** central precocious puberty  **-** PCOS  - Ovarian failure  - Testicular failure | 1/15  1/15  0/15  1/15 | 2/27  2/27  1/27  0/27 | 0.89  0.89  1  0.36 |
| **Death** | 0/15 | 2/27 | 0.53 |

**Footnotes:**

Median values were used to summarize the continuous quantitative variables with extreme values (range) in parentheses. Frequencies were used to summarize the qualitative variables and were reported as "No. (%)".

**Abbreviations:**

*Continuous quantitative variables were compared using the Student t test. Qualitative variables were compared using Fisher's Exact test. Statistical analysis was performed using the open source software R (v3.5.1., 2018, R Core Team, Vienna, Austria). Significance was determined based on p value < 0.05.

F: Female

M: Male

N: normal (2N: 2 times higher than normal)

PCOS : polycystic ovary syndrome

rhGH : recombinant human Growth Hormone

SDS: standard deviation score
